# Caught in travertine: computed tomography reveals the youngest record of *Amphicyon giganteus* from the travertine deposits of Karacalar (late middle Miocene, central Anatolia, Turkey)

**DOI:** 10.1007/s12542-022-00610-0

**Published:** 2022-02-19

**Authors:** Julien van der Hoek, Aslı Karabaşoğlu, Serdar Mayda, Lars W. van den Hoek Ostende

**Affiliations:** 1 Present Address: Department of Earth Sciences, Uppsala University, Villavägen 16, 75236 Uppsala, Sweden; 2 Naturalis Biodiversity Center, P.O. Box 9517, 2300 RA Leiden, The Netherlands; 3 Geological Engineering Department, Afyon Kocatepe University, 03200 Afyonkarahisar, Turkey; 4 Faculty of Science, Department of Biology, Ege University, Izmir, Turkey

**Keywords:** Afyonkarahisar, Amphicyonidae, *Amphicyon giganteus*, 3D reconstruction, Travertine

## Abstract

A computed tomography scan of a travertine slab from the Karacalar Silver Travertine Quarry (Afyonkarahisar Province, Turkey) revealed the presence of an encased partial cranium, partial mandible and three vertebrae. 3D reconstruction of the fossil helped identifying it as *Amphicyon giganteus.* As the travertine caps a section correlated to MN7/8, the specimen represents the youngest record of *Amphicyon giganteus*, the known range previously being limited to MN4 – MN6. This young age is in line with the more advanced morphology of the lower molars.

## Introduction

The Cenozoic geological history of Anatolia has been one of much tectonic activity. Most of current-day Anatolia was shaped by the northward movement of the African and Arabian plates against the relatively stable Eurasian plate. Due to these forces and the more recent pull of the Hellenic trench, the Anatolian block has been pressed westward (McClusky et al. 2000, 2003; Reilinger and McClusky 2011). Because of this tectonic activity, Anatolia is rich in travertine deposits, as tectonic activity results in the formation of travertine from hot springs that occur along active faults (Hancock et al. 1999). Famous examples of these deposits include Kocabaş, where a *Homo erectus* was found in travertine (Kappelman et al. 2008), and Pamukkale, which is a UNESCO-World Heritage Site and tourist attraction.

Travertine provides a suitable fossilization environment, as animal and plant remains can be quickly sealed off. As it consists primarily of calcium carbonate, it is an excellent material for the preservation of bones and teeth. Moreover, the fossil is protected from the acidic properties of rain that might cause corrosion or dissolution, as the first stages of decalcification are primarily focused on the travertine instead of on the fossil itself (Walker 2005). The enclosed fossils can be used to give an age estimate for the travertine (e.g., Erten et al. 2005). Direct dating of the rock is difficult, as the carbonate minerals that make up travertine are often formed at different times, rates, and in different locations (Pentecost 2005). Moreover, radiometric dating with ^14^C or ^234^U/^230^Th, Electron Spin Resonance, and thermoluminescence dating all have age limits that make them unsuited for dating anything beyond the Pleistocene (Dreimanis 1978; Grün 2005; Pentecost, 2005). Of course, if the travertine is part of a larger section, radiometric dating of other layers and palaeomagnetostratigraphy can be used to infer a time of deposition. In the case of the *Homo erectus* from Kocabaş, this proved especially useful, as the Villafranchian fauna from the site per se could not provide as precise a date (Lebatard et al. 2014). However, usually, the enclosed fossils are the only way to date a travertine deposit.

Unfortunately, studying fossils from travertine deposits provides a major logistic challenge. Encased in a hard matrix, it is almost impossible to study the fossils directly. Computed tomography scans can provide a solution. In this paper, we describe a carnivoran skull and mandible preserved in a travertine slab from the Karacalar Silver Travertine Quarry, in the province of Afyonkarahisar around 200 km southwest of Ankara. On the basis of X-ray images, it was hypothesized that the fossil represented a large-sized amphicyonid, although at that stage, a primitive ursid could not be excluded. Therefore, the fossil was reconstructed using computed tomography to provide an accurate identification and description. Apart from our interest in the fossil itself (carnivoran skulls are relatively rare in the fossil record), we hoped that the fossil would allow us to assign an age to the travertine itself. The quarry is situated in the upper part of the Gebeceler Formation. The lower part of that formation yielded a mammalian fauna with *Anchitherium* sp., *Hispanotherium grimmi*, *Caementodon* cf. *caucasicum*, *Micromeryx flourensianus, Triceromeryx* sp., *Hispanomeryx* sp., *Giraffokeryx* sp. nov., and *Sinapospalax* cf. *berdikensis*, allowing a correlation to MN7/8 (Saraç 2003; Mayda et al. 2013). Thus, the maximum age for the Karacalar fossil is set at the end of the middle Miocene.

## Geological setting

The oldest geologic units in the study area are the Paleozoic crystalline metamorphic rocks (Metin et al. 1987). These rocks are mostly composed of marble, calcschist, albite–chlorite–muscovite–quartz schist and meta-conglomerate levels. In addition to these, porphyroid rocks in metamorphics cover large areas in the southwest of Afyon. The upper contact of the Afyon metamorphites is discordantly overlain by the Mesozoic and Miocene units. The Mesozoic units in the area are defined as the Bolkardağı unit (Özgül 1976). These formations are called Tozlutepe (Middle – Late Triassic), Koçakkaletepe (Jurassic – Cretaceous) and Kaledere (Late Cretaceous) (Alan et al. 2007). Neogene units consist of lacustrine environment products, with the Miocene aged Gebeceler Formation and Köroğlu Volcanics consisting of equally aged volcanic rocks transitive in lateral and vertical direction (Seydiler Pyroclastic Member, Kocatepe Lava Member and Karakaya basalt member). These are lahar, recoil deposits, ignimbrite, block-ash flows and trachytic lavas which are products of volcanism.

The Gebeceler Formation exposed on most areas surrounding the Karacalar Village consists of pebble stone, pebbly sandstone, tuff- tuffite clayey limestone at the base, thin-medium bedded marls and transitional lacustrine limestones. The thickness of the unit reaches 345 m (Metin et al. 1987). In previous studies, mammal fossils were mentioned from the unit, dating it to the middle Miocene (Saraç 2003). In addition, 11.6 ± 0.25 my K/Ar age was taken from tuffite levels in volcanoclastics (Besang et al. 1977). There are many travertine quarries in the region, where the lacustrine limestones are mined as natural stones (travertine). In these lacustrine limestones, vertebrate fossil remains, plant fossils, fossil reeds and algae have been identified. These travertine deposits were formed as a result of many inactive faults.

## Material and methods

### Material

**Fig. 1:**
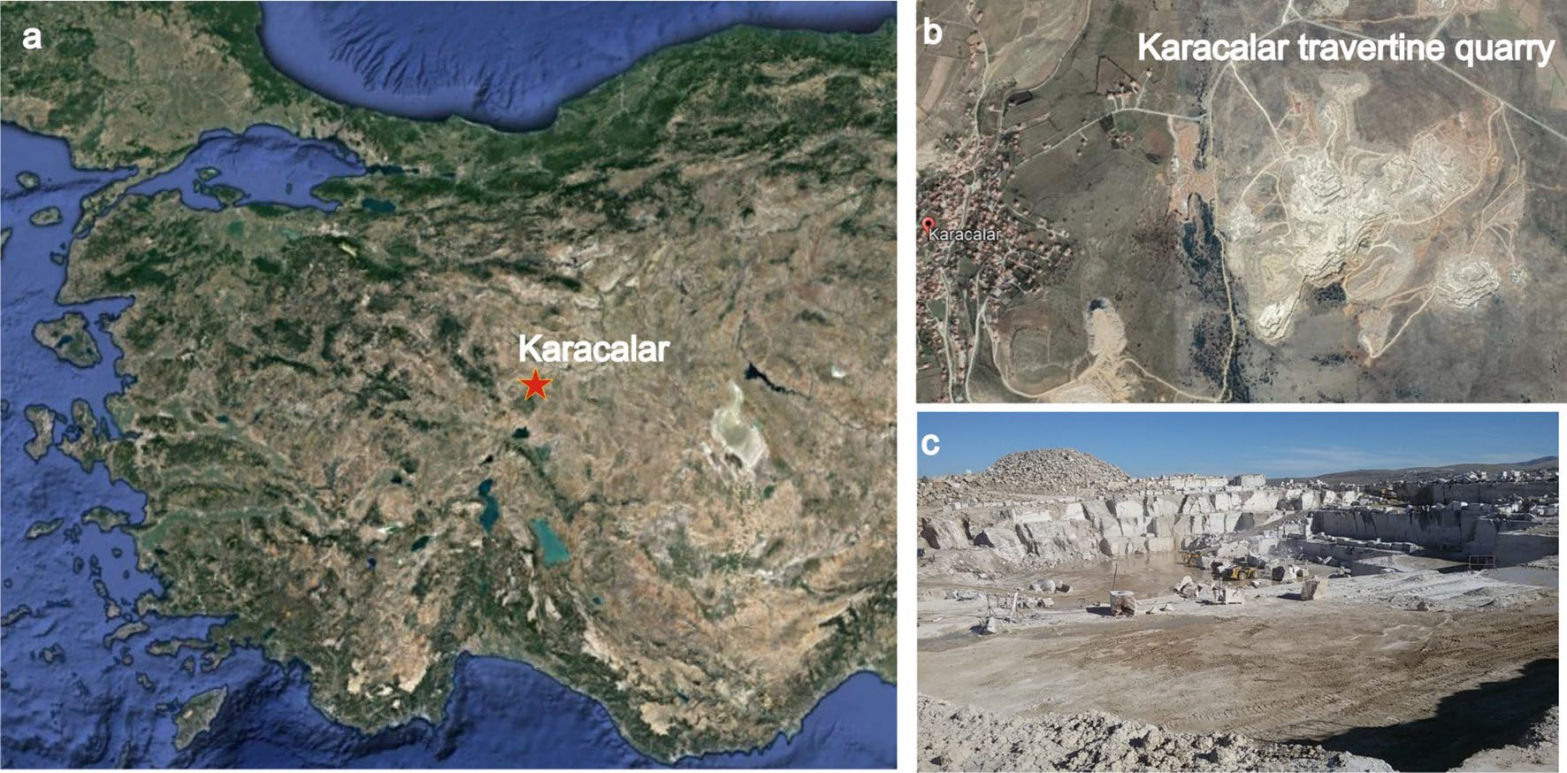
**a**, **b** Location map (enlarged) of the study area. The locality Karacalar is indicated by an asterisk, Sample coordinate: 38° 58′ 44.78″ N, 31° 15′ 58.23″ E. **c** Close-up photograph of the Karacalar travertine field

**Fig. 2:**
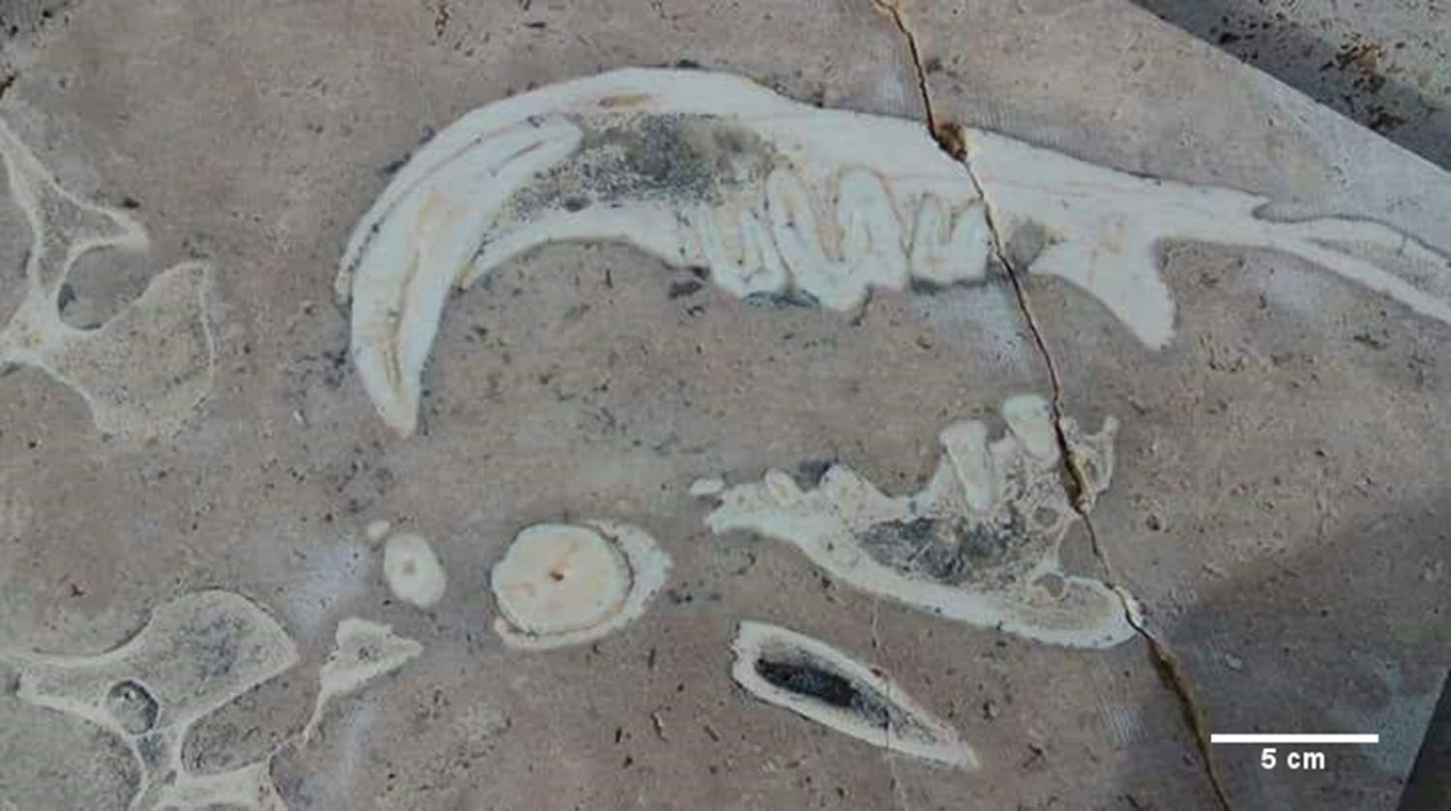
Karacalar travertine slab in situ

The fossil was found in 2018 during quarrying at the *Karacalar Silver Travertine Quarry* at 38° 58′ 44.78″ N, 31° 15′ 58.23″ E (Fig. 1). The travertine was cut by the quarrying operations into a slab that is about 43 cm long, 43 cm wide and 4 cm thick. A mandible, partial cranium and a few vertebrae can be distinguished in the slab (Fig. 2). The studied material is stored in the Afyon Kocatepe University Geology Department under the temporary inventory number Afyon-1. It will later be moved to be stored under a permanent inventory number in the same department.

### Computed tomography scan

The travertine slab was placed into a computed tomography scanner in the Medical Research centre of Afyonkarahisar. 899 pictures were taken of cross sections in three dimensions. The machine used for the CT-scan was the Aquilion Prime with an exposure time of 12 h, 20 min and 57 s at 120 kV. The resulting voxel size was 0.876021 × 1 × 0.876021.

### 3D reconstruction

**Fig. 3:**
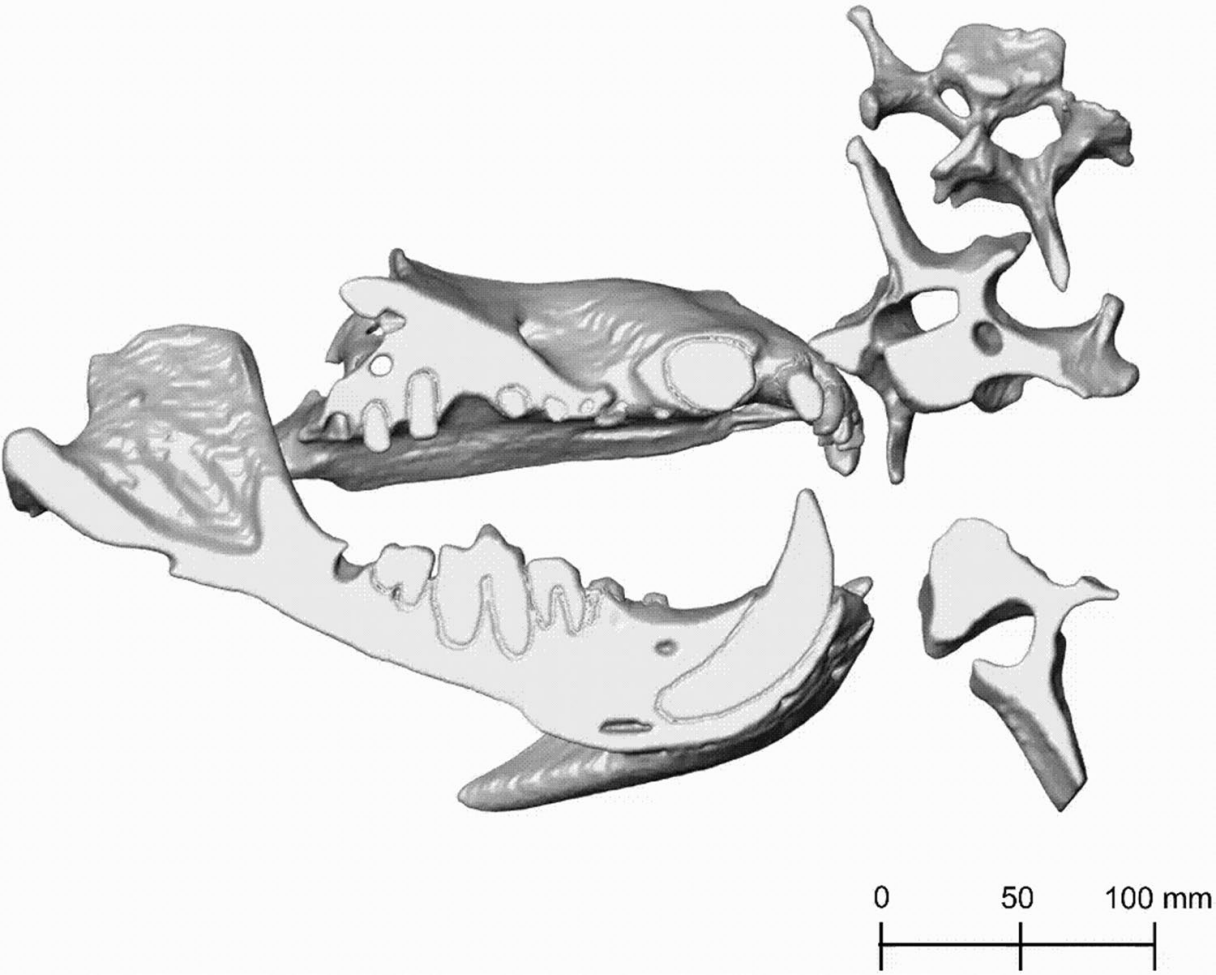
3D reconstruction of the Afyon-1 specimen of *Amphicyon giganteus* in labial view. Scale bar of 100 mm

Avizo 2019.4 was used to segment the fossil and to create a 3D reconstruction by stacking the segmented materials three-dimensionally (Fig. 3). The mandible, cranium and teeth were each segmented using the brush tool to colour each different material by hand. After segmentation, the 3D reconstruction was smoothed by first using *Remove islands* on all connected regions smaller than 15 voxels on all slices. Then, *Smooth labels* was used on size three on the 3D-Volume. The use of *Remove islands*, *Smooth labels* and their tool settings were taken from the tutorial “3.5 Segmentation of 3D images” from the user’s guide for Avizo software (ThermoFisher Scientific 2018).

### Measurements

The dental nomenclature used follows Schmidt-Kittler (1976). Measurements were taken according to the protocol developed by Peigné and Heizmann (2003). The measurements were taken using the measurement tool in Avizo. Measurements were taken with an accuracy of 0.1 mm (Table 1).

**Table 1:** Measurements for the Karacalar specimen of *Amphicyon giganteus* (Afyon-1), with the “ ~ ” symbol denoting an estimated value and a “ > ” symbol denoting a value of a measurement that was cut off

Material	Length	Width	Labial height	Lingual height	Height	Distal width	Trigonid labial length	Trigonid lingual length	Talonid width	Paraconid height	Protoconid height	Metaconid height
I1	9.3	11	14	10								
I1 sinistral	8.8	~ 11.8	~ 13.3	7.7								
I2	11	14	22	17								
I2 sinistral	10.3	15.1	≥ 17.7	17.7								
I3	19.6	16.2	> 13.7	> 15.2								
C	35	29			–							
P1	~ 7.3	~ 4.9			4.3							
P2	~ 13.0	~ 5.3			5.8	~ 5.8						
Diastema C – P1	0											
Diastema P1 – P2	7.8											
i3	8.3	15	11	17								
c	31	> 18.5			43							
p2	12	5.3			4.3	4.9						
p3	16	7.5			6.3	> 8.7						
p4	22				13	11						
m1	36.6	> 13.8					24.7	26.5	> 13.4	13.8	22.7	14.2
m2	24	> 13.7									16	11
Diastema c – p1	–											
Diastema p2 – p3	6.2											
Diastema p3 – p4	1.5											
Mandible	342											

All dental measurements are made of dextral teeth, unless indicated as “sin”. See Peigné and Heizmann (2003) for explanations of the measurements

## Systematic palaeontology

Order **Carnivora** Bowdich, 1821

Family **Amphicyonidae** Haeckel, 1866

Subfamily **Amphicyoninae** Haeckel, 1866

Genus **Amphicyon** Lartet, 1836

Type Species. *Amphicyon major* de Blainville, 1841

***Amphicyon giganteus*** (Schinz, 1825)

See Ginsburg and Antunes (1968) for a synonymy list before 1968.

**Table:** 

	1968	*Amphicyon giganteus* Ginsburg and Antunes (1977), pp. 9–12, 14–19, 24, figs. 1–24, 28, 29, 31
?	1977	*Amphicyon giganteus carnutense* Ginsburg and Antunes (1977), p. 341
?	1984	*Amphicyon giganteus* de Beaumont (1984), p. 81, pl. 1, figs. 1–5
?	1989	*Amphicyon giganteus carnutense* Ginsburg (1989, 2000), p. 103, figs. 1–4
?	1996	*Amphicyon giganteus* Viranta, p. 16, fig. 3
	1998	*Amphicyon giganteus* Morales, Pickford, Soria and Fraile (1998), p. 32, fig. 7
	2000	*Amphicyon giganteus* Ginsburg (1989, 2000), p. 36
	2003	*Amphicyon giganteus* Morales, Pickford, Fraile, Salesa and Soria (2003), p. 191, pl. 4, figs. 4–6
	2006	*Amphicyon giganteus* Peigné, Salesa, Antón and Morales (2006b), p. 365, pl. 2, figs. 1–15
	2018	*Amphicyon giganteus* Bastl, Nagel, Morlo and Göhlich (2018), p. 4, fig. 2
	2019	*Amphicyon giganteus* Morlo, Miller, Bastl, Abdelgawad, Hamdan, El-Barkooky and Nagel (2019a), p. 739, fig. 4
	2020	*Megamphicyon giganteus* Siliceo, Morales, Antón and Salesa (2020), pp. 225, 227–232, figs. 1–7

*Holotype*: Left M1 from Avaray (Loir-et-Cher), stored at the Museé d’Orléans. Figured by Cuvier (1824, pl. 193, fig. 20); Mayet (1908, p. 83, fig. 24, p. 211, fig. 68, pl. 8, fig. 7); Kuss (1965, p. 68, fig. 42).

*Range*: MN4 – MN7/8.

*Occurrence*: Anatolia, Europe, Egypt, Namibia.

*Material*: Encased partial cranium and mandible, partially cut off on the side of the slab, Afyon-1.

## Description

### Lower dentition

**Fig. 4:**
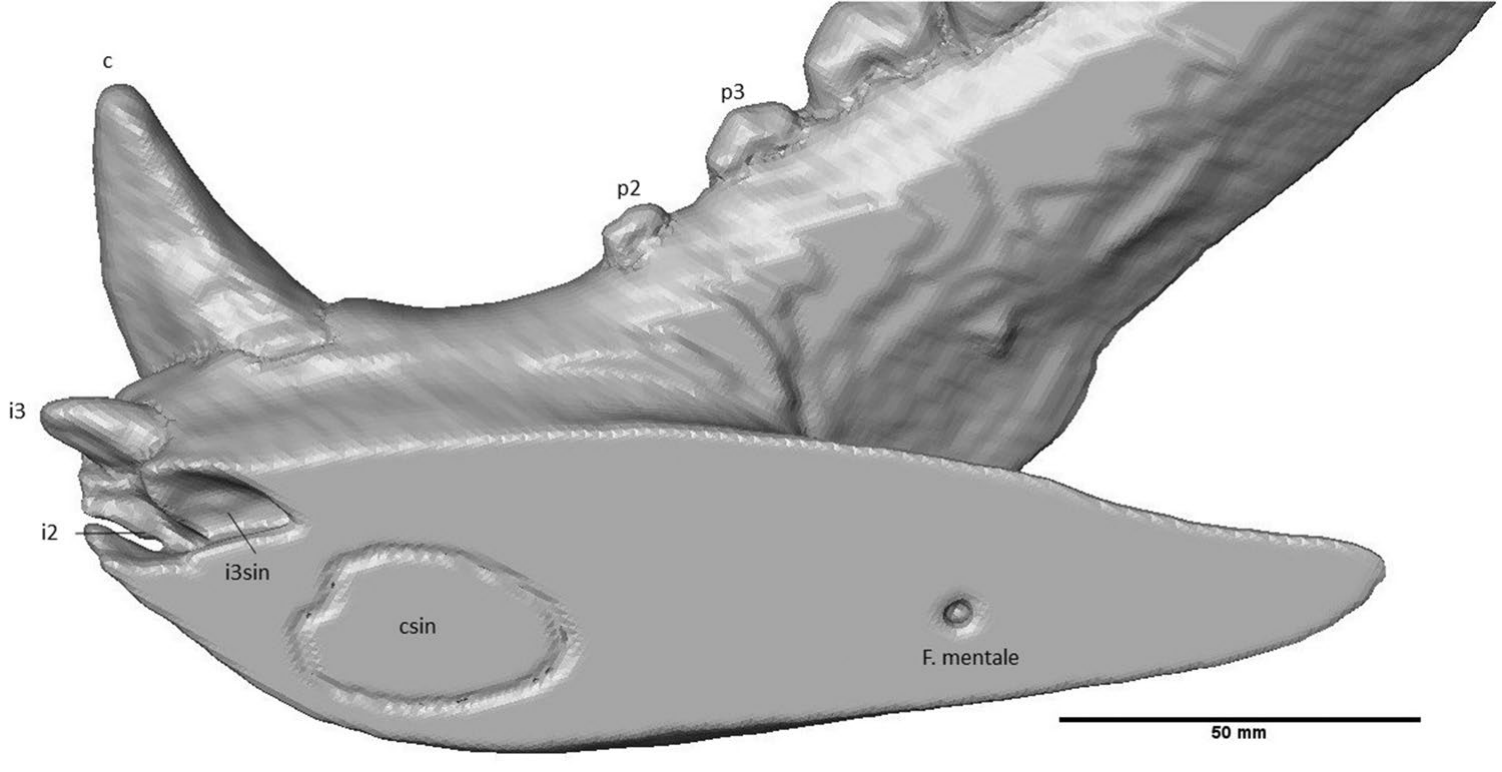
i3, c and alveoli of the Afyon-1 specimen of *Amphicyon giganteus* in lingual view, cranium and vertebrae excluded from the reconstruction. Scale bar of 50 mm

**Fig. 5:**
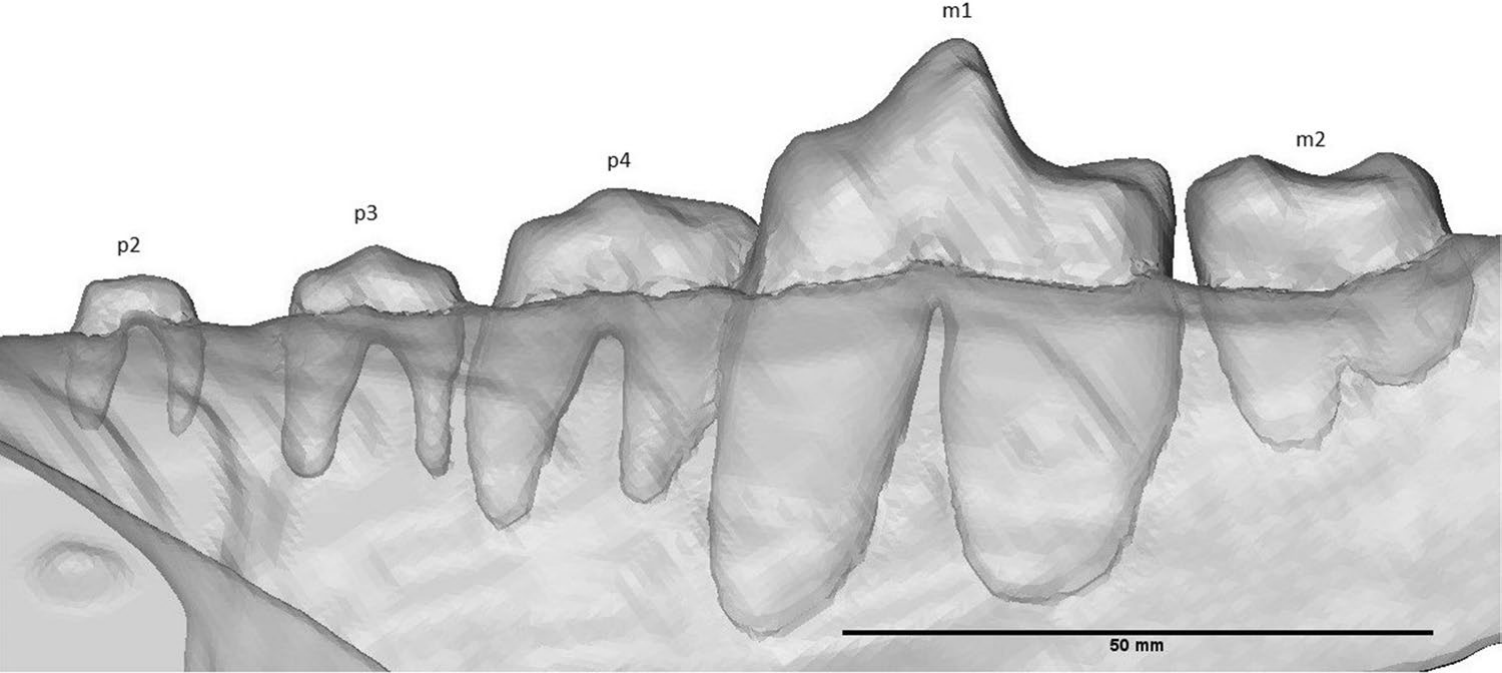
p2 – m2 of the Afyon-1 specimen of *Amphicyon giganteus* in lingual view, roots were made visible by setting transparency to 0.5. Scale bar of 50 mm

**Fig. 6:**
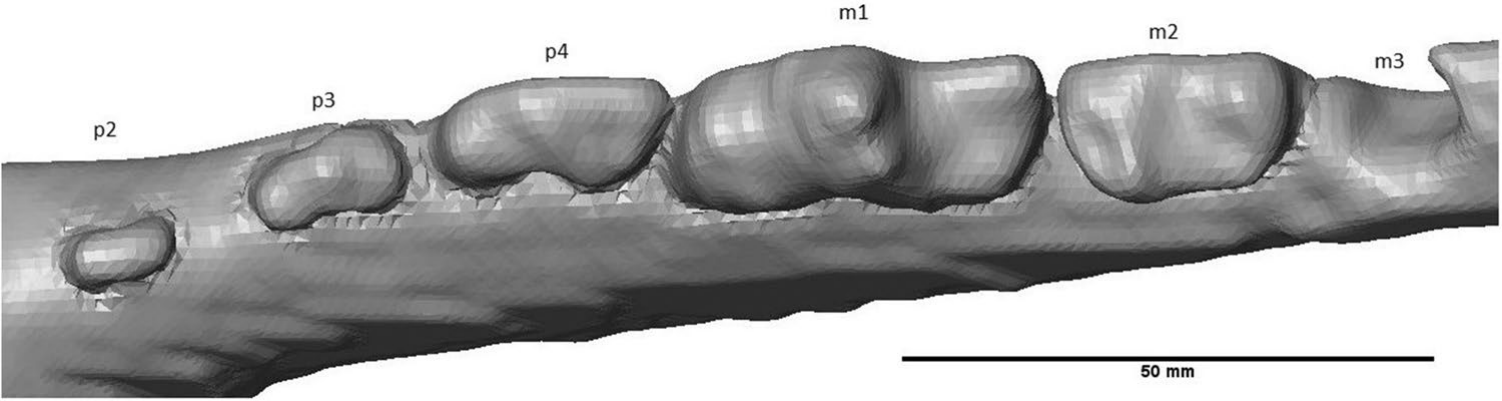
p2 – m2 of the Afyon-1 specimen of *Amphicyon giganteus* in occlusal view, cranium excluded from the reconstruction. Scale bar of 50 mm

On the right mandible, one procumbent i3 was preserved, which is longer than it is wide (Fig. 4). The mesial side of i3 is concave; the distal side is convex. The root of i3 is convex on the mesial side, while on the distal side of the crown, the root starts out convex tapering to the right, but the lower end is flattened. There is a clear gap between i3 and c. The canine is robust, recurved and has a ridge on the lingual side, which extends from the base to the tip of the crown, due to angular cutting of the slab. Diastemata are of variable length. While a large diastema separates the canine from the p2, diastemata between p2 – 4 are smaller. The p1 and its alveolus are absent (Fig. 5). The mesial roots of p2 and p3 are thicker than their distal roots. The p2 is small, lingually placed and double rooted. It is oval in occlusal view and has one cusp (Fig. 6). From the p3 onwards, the teeth are partially cut off at a diagonal angle on the labial side by the surface of the slab. This affected mostly the crowns of p3, p4, m1 and m2, although the roots are more completely preserved. The p3 is much more robust than the p2. It is double rooted and has an accessory tubercle distally from the main cusp. The p4 is much more robust than the p3, the size difference has a similar ratio as the difference between p2 and p3. The p4 is double rooted and has a more robust distal accessory tubercule than the p3. The m1 is a robust tooth, which is cut off on the labial side. It is labiolingually flattened. It has a strong, high paraconid. The protoconid is high with a metaconid present as a small cusplet in the flank and near the base of the protoconid. The broad talonid has a large labially placed hypoconid and a reduced entoconid. The talonid basin is widest behind the metaconid and protoconid. The mesial root is thinner than the distal root, with a ratio of about 3:5. The m2 is about 66% smaller than the m1. It appears to be sub-rectangular in occlusal view; the labial side is cut off. The m2 has a reduced paraconid; metaconid and protoconid are of about equal size, forming a ridge across the trigonid. The talonid is dominated here by the hypoconid as well. The entoconid curves slightly along the posterolingual corner of the talonid. The roots have a similar relative thickness as in the m1. Behind the m2, the alveolus of a single-rooted m3 is preserved.

### Mandible

Two alveoli belonging to the roots of incisors are situated mesially to the dextral i3. The first alveolus in order is in line with the tooth row and is slightly smaller than the alveolus of i3, which suggests that it is an i2. The second alveolus is placed more towards the sinistral side than i2 and rotated at a similar angle as i3. Due to this placement and angle, it is interpreted as a sinistral i3. A root of a canine is visible on the sinistral side of the fossil, but it does not match the angle of the dextral canine and seems to be situated less ventrally in the mandible. It is interpreted here as the root of the sinistral canine.

**Fig. 7:**
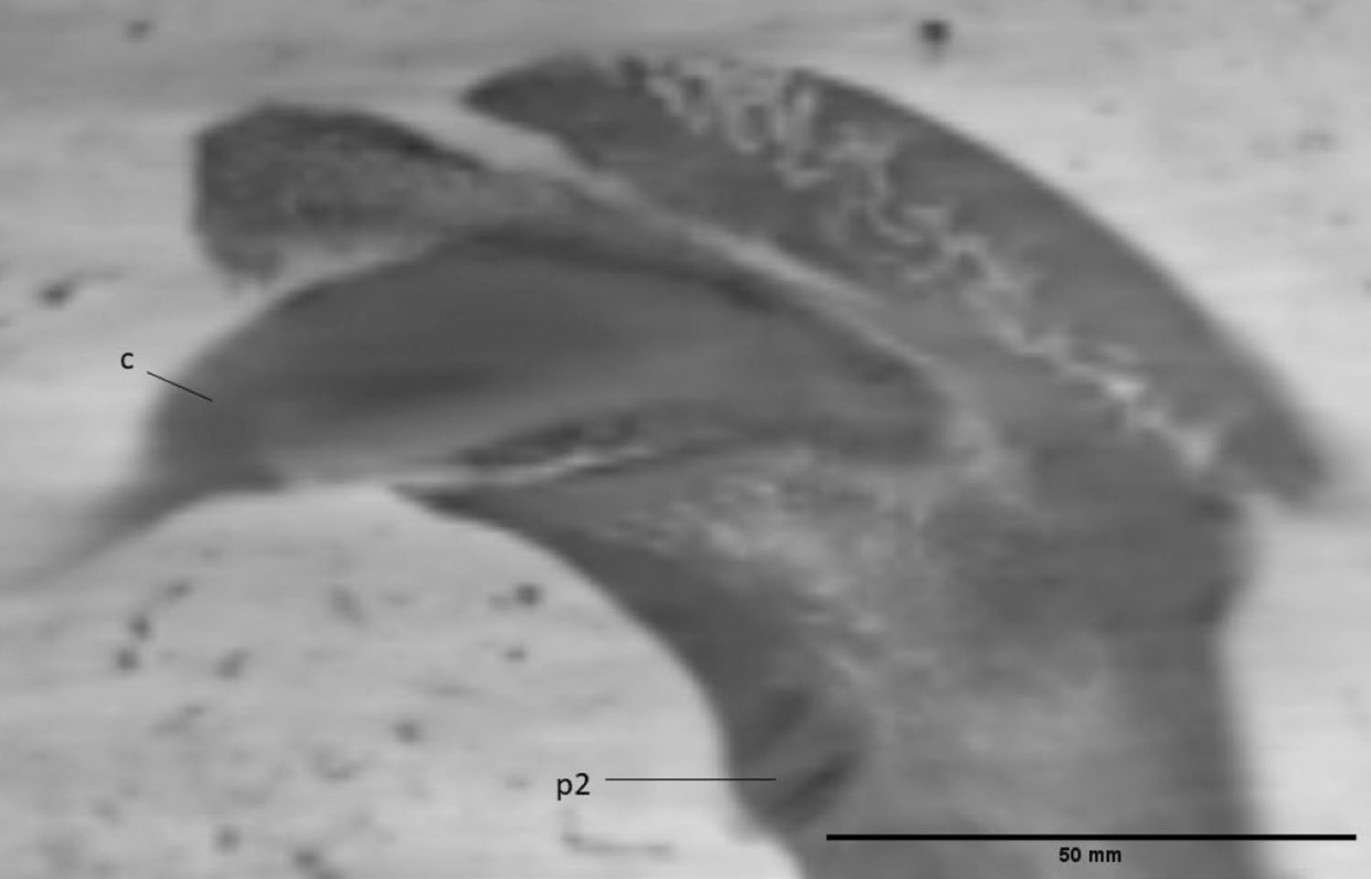
Anterior part of the crushed mandible of the Afyon-1 specimen of *Amphicyon giganteus*. Scale bar of 50 mm

**Fig. 8:**
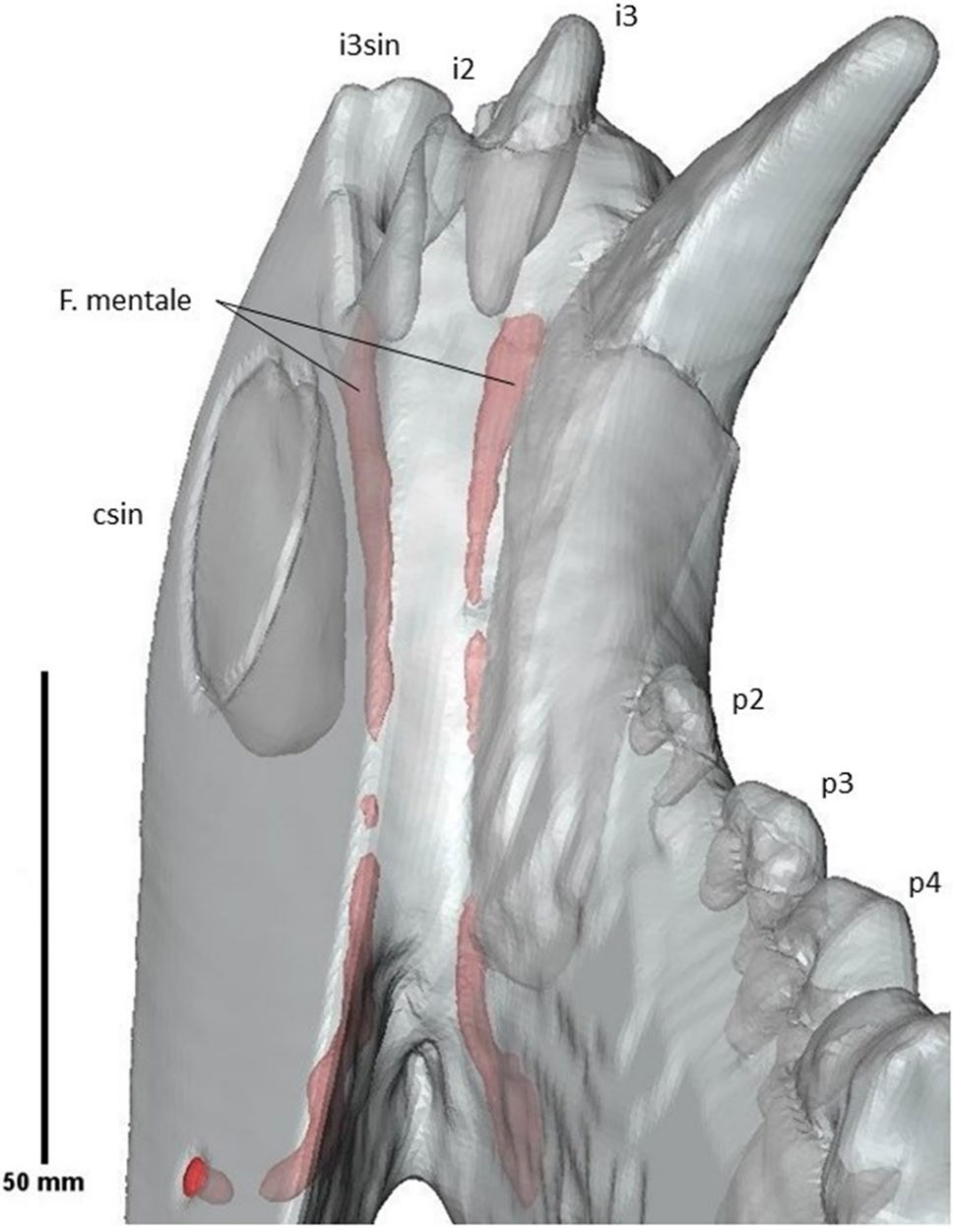
Long, fragmented canals of the mental foramina (in red) of the Afyon-1 specimen of *Amphicyon giganteus*. The canals were made visible by first segmenting the tunnels, and then setting transparency to 0.5, vertebrae and cranium excluded, lingual view. Scale bar of 50 mm

A porous structure can be seen throughout the mandible mesial to the canine and i3 (Fig. 7). The presence of this structure suggests some crushing occurred in this location during fossilization. The anterior foramen mentale is preserved both on the right and the left side, below the i3 and the alveolus of that element, respectively. It aligns via a long internal canal with mental foramina that can be seen as an oval-shaped opening on the dextral side of the fossil underneath the proximal root of the p2 and distal root of the p3 and as a smaller opening on the sinistral side underneath the p3 (Fig. 8). On the labial side, the mesial border of the ascending ramus has been preserved. The condyle is cut off on both the lingual and labial side. It is situated above the tooth row.

### Upper dentition

**Fig. 9:**
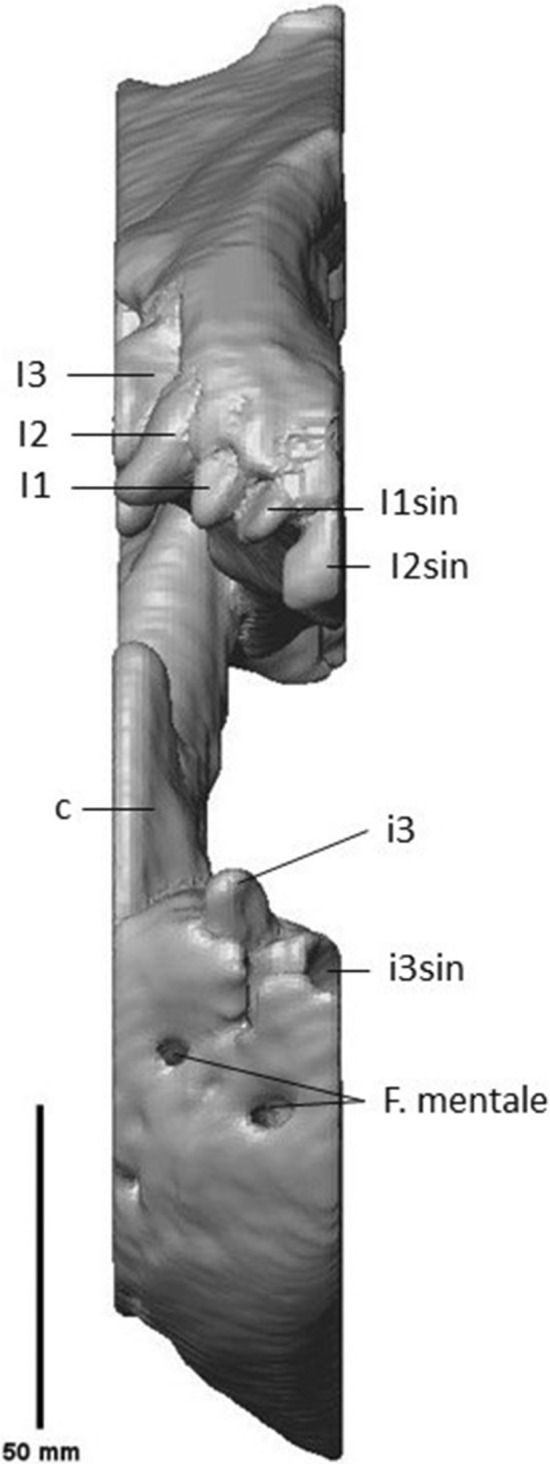
Partial cranium and mandible of the Afyon-1 specimen of *Amphicyon giganteus* in anterior view, vertebrae excluded from the reconstruction. Scale bar of 50 mm

**Fig. 10:**
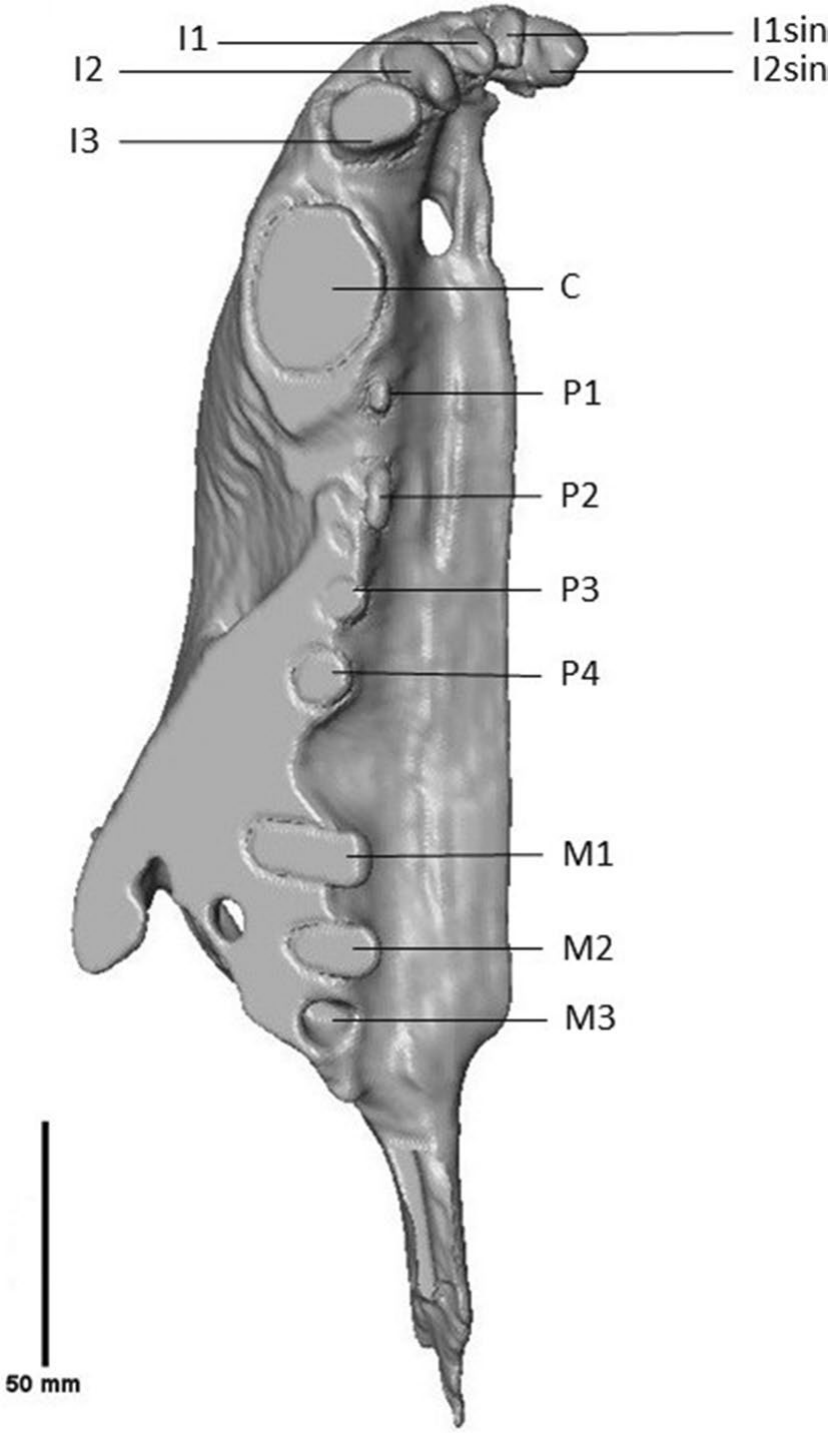
Partial cranium of the Afyon-1 specimen of *Amphicyon giganteus* in ventral view, mandible and vertebrae excluded from the reconstruction. Scale bar of 50 mm

Only part of the sinistral side of the specimen was preserved due to the angle in which the travertine slab was cut. In this way, the sinistral I1 and I2 are preserved next to the anterior part of the dextral dentition (Fig. 9). The incisors become progressively larger in size and are single rooted. They are notably robust. The dextral I1 is convex on the mesial side, but flattened on both the distal side and along the labiolingual axis. This morphology differs notably from the sinistral I1, which appears damaged due to the erosion of the front of the fossil also seen in the displaced lower canine. It is similarly sized, but has an elongated and narrow apex on the labiolingual axis. Its root is exposed. The dextral I2 is larger than I1 and curves towards I3. It is narrow and elongated, convex mesially and concave distally. The apex is a rounded edge. The sinistral I2 is cut off by the travertine slab. On its lingual face, the tooth curves mesially. Its apex is rounded. I3 is a caniniform, robust tooth. The tooth is cut off on the labial side. The root of the tooth is curved. Only the root and the base of the crown of the upper canine have been preserved; based on their sizes, the C can be interpreted as a robust tooth. The root narrows dorsoventrally in a lingual direction from the base of the crown to the tip of the root. P1 is a small, unicuspid and single-rooted tooth, laying in close proximity to the canine (Fig. 10). P2 is double rooted and unicuspid, oval in shape from an occlusal view. P3, P4, M1 and M2 are cut off at such an angle that mostly the roots are preserved. Only a small fragment of the crown is preserved in these teeth. P3 has partial preservation of two roots, but P4 to M2 only have one root with a small fragment of the crown preserved. A gap is present between the root of P4 and the root of M1, as a result of the two labial roots of the P4 having been cut off. An alveolus can be seen distally to the last root of the maxilla, which is interpreted as the alveolus of the root of M3.

### Cranium

Part of the bony palate can be distinguished, with a palatine fissure. The most anterior part of the zygomatic bone is preserved. The most posterior part of the skull preserved is part of the pterygoid bone. The back of the cranium seems to be damaged. In anterior view, the nasal aperture and the infraorbital foramen can be distinguished. Posteriorly, the infraorbital foramen continues into a narrow infraorbital canal that widens towards the end. The nasal cavity is large and deep and can be seen in lingual and anterior views.

## Comparison

Both the premolars and the metaconid of m1 are clearly in reduction, which would suggest an adaptation to hypercarnivory similar to genera such as *Tomocyon* or *Agnotherium* (Morlo et al. 2019a, b; Viret 1929). Unlike *Agnotherium*, however, the metaconids on the m1 and the premolars are not as extremely reduced (Morlo et al. 2019a, b). *Tomocyon* also does not possess a metaconid on the m1. It has a taller talonid on the m1 than Afyon-1. The p4 of *Tomocyon* is much taller than the p4 of Afyon-1 (Viret 1929).

Based on the robust dental elements, the diastemata between the premolars and the presence of an entoconid on the m1 (Viranta 1996), the Karacalar specimen can safely be attributed to the genus *Amphicyon*. Within the genus, it shows the greatest similarity with *A. major* and *A. giganteus*, which are morphologically similar, with *A. giganteus* generally being larger (Viranta 1996).

No upper dentition behind the P2 was preserved. These teeth, especially the upper carnassial and molars, are often used in identifying Amphicyonidae and separating *Amphicyon giganteus* from *A. major* (Gürbüz 1974; Peigné et al. 2006b; Jiangzuo et al. 2019). Another characteristic often used in separating the taxa is size, but both *A. giganteus* and *A. major* display significant variation in size, which can largely be explained by sexual dimorphism, as males are larger than females in both species (Viranta 1996). These size variations were estimated to be up to 20% for *A. major* and slightly over 30% for *A. giganteus* (Ginsburg and Antunes 1968).

**Fig. 11:**
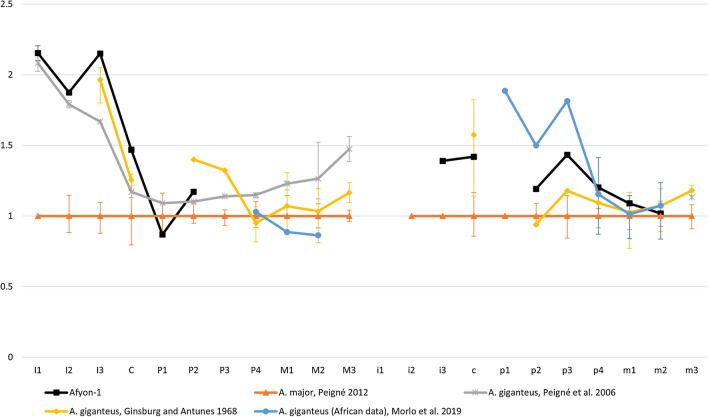
Afyon-1 dental length compared to *Amphicyon major* and *A. giganteus*, scaled relative to the material of *A. major* from Sansan. Error bars represent the size ranges of the teeth. The data were taken from Ginsburg and Antunes (1968), Peigné (2012), Morlo et al. (2019a, b), Peigné et al. (2006a, b)

Whereas absolute size needs to be used with caution, the relative size of the anterior dentition differs markedly between the two species, the upper incisors, lower premolars and canines being relatively smaller in *Amphicyon major*. This size difference in anterior dentition is clearly visible when comparing data from Bergounioux and Crouzel (1973) and Peigné (2012). In Fig. 11, we plotted the lengths of the elements of the Karacalar specimen against that of *A. major* from its type locality Sansan, setting the value of the latter to 1. The measurements used for this plot are presented in Appendix I1 and II Appendix 2 for the upper and lower dentition, respectively.

The Karacalar specimen fits the descriptions of *Amphicyon giganteus* by Ginsburg and Antunes (1968) in their overview of the species based on material from France, Germany, Austria, Switzerland and Portugal. When looking at the m1 of the Karacalar specimen, the talonid basin is not at its largest behind the posterior crest of the hypoconid, which would be a characteristic of *A. major*. The tooth is also more robust, with stronger cingulids. When looking at the m2, the tooth resembles the occlusal view of the m2 from Captieux, but no clear morphological differences separate the m2 from that of *A. major*.

The dentition of *Amphicyon giganteus* was described by Viranta (1996), based on material from France and Spain, with additional data from Ginsburg and Antunes (1968). Viranta mentioned a double-rooted m3 for the material from Neuville-aux-Bois (MN3). This material was later redefined as belonging to *A. laugnacensis* (Ginsburg 1999). In younger material from Arrisdrift, Moghra and Arroyo del Val, the last lower molar is described as single rooted (Morales et al. 1998, 2003; Peigné et al. 2006b; Morlo et al. 2019a, b). The m3 from the Karacalar specimen is single rooted as well. Thus, the m3 of *A. giganteus* is single rooted. Viranta (1996) also indicated the presence of a cingulid around m1. A cingulid is identified in material from Moghra (Morlo et al. 2019a, b), but not in other material (e.g., Morales et al. 2003; Bastl et al. 2018; Siliceo et al. 2020), and is also absent in the Karacalar specimen. The other characteristics can be identified in both the Karacalar specimen and most specimens identified as *A. giganteus*.

PQAD 1520, a mandible from Arrisdrift, Namibia, was placed in *Amphicyon giganteus* as opposed to *A. major* based on its larger premolars, lack of diastemata between the premolars and its overall large size (Morales et al. 1998, 2003). The *Amphicyon giganteus* material from Moghra, Egypt shows great similarity to the material of Arrisdrift (Morales et al. 1998, 2003; Morlo et al. 2019a, b). CUWM 53 shares the double-rooted p2, lack of diastemata, low metaconid and well-developed hypoconid on m1, an m2 with a paraconid and the presence of a single-rooted m3 with PQAD 1520 (Morlo et al. 2019a, b). As such, CUWM 53 shows similar affinities with the Karacalar specimen as PQAD 1520. The larger premolars in the African material can also be seen in the Karacalar specimen and fits in the range of *A. giganteus* (Viranta 1996). In contrast to the Arrisdrift and Moghra specimens, diastemata are present between the premolars of the Karacalar specimen, but they are smaller than those recorded for the *A. major* material from Sansan (Bergounioux and Crouzel 1973). This difference in diastema size is also used in some cases as a characteristic separating *A. giganteus* from *A. major* (e.g., Morales et al. 1998, 2003; Morlo et al. 2019a, b).

*Amphicyon giganteus* material from La Barranca (Arroyo del Val area, MN6) has incisors, canines and premolars that resemble the Karacalar specimen. Peigné et al. (2006b) stated that these teeth are not indicative of *A. giganteus* in themselves and based their identification on the morphology of the P4. However, the La Barranca anterior dentition is close in length to the Karacalar specimen and, more importantly, shows a similar relative size to the typical *A. major* (Fig. 11). According to Peigné et al. (2008), *Amphicyon major* and *A. giganteus* also have different ratios of the length of the p4 to the length of the m1. For *A. major*, this range is 0.48 – 0.54, while it is 0.51 – 0.60 for *A. giganteus* (Peigné et al. 2008). The p4/m1 ratio of the Karacalar specimen is 0.59, fitting well into the range of *A. giganteus*.

Material from Gračanica (Bosnia and Herzegovina, MN5) includes a dextral p4 – m2 belonging to *Amphicyon giganteus*, with a fragmentary p4 (Bastl et al. 2018). The molars have a similar length as the Karacalar specimen. Other commonalities are the distal accessory cusp in p4, the robustness of the crown and roots of m1, a metaconid that is close to the protoconid in m1 and the occlusal outline of m2.

Recently, material of *Amphicyon giganteus* was described from the MN6 locality of Carpetana, Spain (Siliceo et al. 2020). The material agrees with the Karacalar specimen for the morphology of the premolars are the diastemata between p2–p4, the p3 and p4 with distal accessory cuspids and large p4. The morphology of the molars is similar as well, with a labiolingually flattened m1 with a short paraconid and the metaconid close to the protoconid, a large hypoconid on m1 and m2 and reduced paraconid on m2.

Given the similarity of the Karacalar specimen to material previously described as *Amphicyon giganteus*, there can be little doubt that it belongs to that taxon. The only notable difference is the absence of diastemata in the African material, but, as we have not seen those specimens, that falls outside the scope of this paper. The material from Karacalar is identified as *A. giganteus* based on its size, double-rooted p2, robust incisors and canines, m1 with a narrow talonid basin and metaconid in close proximity to the protoconid, large premolars and small diastemata between them.

Based on previously published material, a number of evolutionary trends can be observed within the species for a number of these structures in the lower dentition (Ginsburg and Antunes 1968). Plesiomorphic characteristics include a more hollowed-out talonid in the m1 and an m2 that is reduced distally and has a strong paraconid. More derived specimens have an m1 with a talonid with a less hollowed-out interior that shrinks gradually and a more rectangular m2, with a barely indicated paraconid (Ginsburg and Antunes 1968). In the Karacalar specimen, these characteristics appear to be in line with a more derived animal, as the talonid of m1 has a narrow, shallow basin and the m2 is sub-rectangular with a very small paraconid. This fits well with Karacalar being the youngest occurrence of *Amphicyon giganteus*.

## Remarks

Kuss (1965) erected the genus *Megamphicyon* for the species *giganteus*, separating it from *Amphicyon* on the basis of size and details in the dentition. This generic distinction was not recognised by subsequent authors (e.g., Morales et al. 2003; Peigné et al. 2006b; Morlo et al. 2019a, b), but, recently, Siliceo et al. (2020) reinstated the genus *Megamphicyon*. As we noted above, the differences in dental morphology between *Amphicyon giganteus* and *A. major* are very small. Siliceo et al. (2020), who included well-preserved postcranial elements in their study, state in their introduction that there “are enough morphological differences to support a generic separation”. However, in their descriptions and conclusions, they stress the similarity with *A. major*. As differences seem to be minor, and size is in itself not a very suitable character to distinguish genera, we prefer to keep the traditional classification with both species in *Amphicyon*, pending a revision of all species of that genus.

Because of the overlap in both geographic and stratigraphic ranges between *Amphicyon major* and *A. giganteus*, Siliceo et al. (2020) suggested that the two amphicyonids should have occupied different niches and may even have lived in different ecosystems. Their body mass estimates of ∼ 150 kg for *A. major* and ∼ 600 kg for *A. giganteus* certainly suggest that the latter was capable of handling larger prey. However, the body mass of *A. giganteus* may be severely overestimated due to the use of a formula for calculating body mass based on tibial measurements from Figueirido et al. (2011), which is not a very accurate way to estimate body size (Siliceo et al. 2020). Whereas the molar morphology of the two species is very similar, the anterior dentition of *A. giganteus* is strongly enlarged compared to that of *A. major*. The exact function of this enlargement, which mostly concerns the incisors, is unknown, but it presumably finds its functionality in the niche differentiation as suggested by Siliceo et al. (2020).

The Karacalar specimen was found in travertine above a layer dated to MN7/8 (Mayda et al. 2013), providing a minimum age for the *Amphicyon giganteus* occurrence. So far, the youngest finds of the species were all dated to MN6 (La Capetana, La Barranca), making the Karacalar specimen the youngest representative to date. The advanced evolutionary stage of the specimen is in line with it being the youngest find of the species. As a consequence, *Amphicyon giganteus* appears to have survived longer in Anatolia after it disappeared from Europe, where there is no record after MN6. No *A. giganteus* material has been found previously in Anatolia, but *A. major* has been identified in the Çandir and Paşalar localities dated to MN5–MN6 (Mayda et al. 2015). In strata below the Karacalar travertine, a large metapodial of an amphicyonid was found (Mayda et al. 2013), which, given the discovery of the Karacalar skull, may also belong to *A. giganteus*. A possibility is that *A. giganteus* being present in Anatolia is part of the greater pattern of migration of Amphicyonidae from Europe into Asia (Peigné et al. 2006a). While a migratory route through southern Europe into Asia has been suggested by Jiangzuo et al. (2019), it was deemed less likely by the authors than a more northwards migratory pattern. While the Asian *Amphicyon zhanxiangi* bears a resemblance to *A. giganteus* (Jiangzuo et al. 2019; Sun et al. 2021), the material is from MN5 – MN6 (Sun et al. 2021), older than the material of *A. giganteus* presented here. Therefore, based on current evidence, it is likely that Anatolia acted as a last refuge for *A. giganteus*. The reason for this southward retreat and the possible replacement of *A. major* requires further understanding on the ecological differentiation between the two *Amphicyon* species and the palaeoenvironmental changes in Anatolia at the end of the middle Miocene.

## Conclusion

The specimen found encased in travertine from Karacalar is identified as *Amphicyon giganteus*, based on its size, double-rooted p2, robust incisors and canines, m1 with a narrow talonid basin and metaconid in close proximity to the protoconid, large premolars and small diastemata between them*.* It represents a more advanced form of *A. giganteus*, based on the more derived morphology of the m1 and m2, in line with the specimen being the youngest representative of the species thus far, based on the age of the deposits directly underlying the travertine. The find suggests that *A. giganteus* found refuge in Anatolia at a time when the species had already disappeared from Europe.

## Data Availability

All data and materials are available through contacting the corresponding author.

## Appendix 1

See Table

**Table 2:** All upper dental lengths used for Fig. 11

Source	Taxon	Specimen	I1	I2	I3	C	P1	P2	P3	P4	M1	M2	M3
This paper	*A. giganteus*	Afryon-1 dextral side	9.3	10.6	19.6	35.1	~ 7.3	~ 13.0					
		Afyon-1 sinistral side	8.8	> 10.3									
Ginsburg and Antunes (1968)	*A. giganteus*	Munich, Stig Museum n ° 13,570											14.7
		Basel, SO (2) 6660				29							
		Basel, SO 360								24.9			
		Graz, Inst. geol								29			
		Graz, Joanneum								27.5			
		Graz, Inst. geol										22	
		Graz, Inst. geol										22.3	
		Basel, SO 4212			18.6								
		Basel, SO 2793						15.5					
		Basel, SO 1700							16.6				
		Basel, SO 874								26.4			
		Basel, SO 4460								27			
		Basel, SO 1274								23.5			
		Museum Paris, Ba. 9								30.6			
		Museum Paris, Ba. 10								29.4			
		Museum Paris, Ba. 11								27.5			
		Basel, SO 1275									24.4		
		Basel, SO 6587									26.1		
		Basel, SO 3338									21.3		
		Museum Paris, Ba. 14									24.1		
		Museum Paris, Ba. 15									26.8		
		Basel, SO 873									24	19.7	
		Basel, SO 2461 a										16.7	
		Fac. Sc. Lyon				31							
		Musée d'Orléans										32	
		Musée d'Orléans											24.6
		Unspecified (Loir-et-Cher)			18.7								
		Unspecified (Loir-et-Cher)										23.5	
		Museum Paris, Fs. 2			16.4								
		Beauvais, coll. Levé, n° 1										22.0	
		Beauvais, coll. Levé, 11° 5											16.6
		Basel, Rd. 137									29		
		Basel, MM. 2034									28		
Peigné (2012)	*A. major*	Sa1				28.2	9.8			31.7	28	22	
		Sa2								30.9	25.5		
		Sa3	4.2		8.8	20.5		10.5	11.7	26.35	21.7	19.3	
		Sa4											
		Sa4-5					9	12	13.1	30.35	25	20.5	
		Sa844	4.2	5.5	9	19.65	7.5	10.7	12.85	26.4	22.15	18.9	13.4
		Sa28								26.7			
		Sa30										22.4	
		Sa31											12.9
		Sa32											14
		Sa22				21.2							
		Sa19				28.1							
		Sa15				26.8							
		Sa16				26							
		Sa23				19							
		Sa18,21				25.7							
		Sa9			8								
		Sa10			8								
		Sa11			9.8								
		Sa12			9.8								
		Sa13			10								
		Sa14			10								
		Sa6		5									
		Sa7		6.5									
		Sa3608			8.6								
Morlo et al. (2019a, b) (African data)	*A. giganteus*	DPC 14532/1a								29.6			
		DPC 5426a									21.7		
		DPC 8981a										17.8	
Peigné et al. (2006a, b)	*A. giganteus*	AR-001								29.4			
		LB-3	8.5										
		LB-4	9										
		LB-6		10.0									
		LB-5		10.3									
		LB-8			15.1								
		LB-7			15.3								
		LB-1				29.0							
		LB-25				27.0							
		LB-2				29.6							
		LB-9					9.2						
		LB-10						12.2					
		LB-11							14.3				
		LB-15								33.2			
		LB-12								33.4			
		LB-21								32.4			
		LB-16									30.4		
		LB-13									30.5		
		LB-24									29.0		
		LB-22									30.4		
		LB-14										23.1	
		LB-23										31.4	
		LB-17										23.7	
		LB-19											18.6
		LB-18											21.0

Measurements from the Afyon-1 specimen were compared with specimens from Ginsburg and Antunes (1968), Peigné (2012), Morlo et al. (2019a, b), Peigné et al. (2006a, b). The inventory number is given unless not stated; otherwise, the museum or locality where the specimen originates from is stated. All measurements in mm

2.

## Appendix 2

See Table

**Table 3:** All lower dental lengths used for Fig. 11

Source	Taxon	Specimen	i1	i2	i3	c	p1	p2	p3	p4	m1	m2	m3
This paper	*A. giganteus*	Afyon-1 dextral side			8.3	31		11.9	15.8	21.7	36.6	23.5	
		Afyon-1 sinistral side											
Ginsburg and Antunes (1968)	*A. giganteus*	Munich, Stig Museum n ° 13,570									32.5		
		Munich, Stig Museum n° 13,565											21.5
		Basel, SO 6521								18.2	35	25	
		Basel, SO 282									33.5		
		Museum Paris, Ch. 1									35.6		
		Musée d'Orléans										21.8	
		Basel, SO 4377									33.4		
		Basel, SO 3424									39.2		
		Basel, SO 3551									25.9		
		Basel, SO 3525										27.0	
		Wien, geol. Bundestalt						9.4	13	18	31		
		Fac. Sc. Bordeaux										27.5	
		Basel, SO 3339								18.7			
		Museum Paris, Ba. 8								20.6			
		Basel, SO 4906								21.9			
		Basel, SO 742									34.5		
		Basel, SO 6729									35		
		Museum Paris, Ba. 18									34.0		
		Basel, SO 6589										24.8	
		Basel, SO 1863										25.9	
		Museum Paris, Ba. 21										20.5	
		Fac. Sc. Lyon, 11° 879								20.8	36		
		Fac. Sc. Lyon									33.3	23.5	
		Fac. Sc. Lyon									38	27	
		Musée d'Orléans				39.9							
		Unspecified (Loir-et-Cher)									38.2	27.0	
		Unspecified (Loir-et-Cher)										27.1	22.9
		Beauvais, coll. Braillon									35.8		
		Museum Paris, Fs. 4									36.1		
		Beauvais, coll. Levé, n ° 54				25.0							
		Fac. Sc. Lisboa				38.5							
Peigné (2012)	*A. major*	Sa844		4.9	6	18.75	5.3	9.3	10.8	16.5	30.35	21.35	17.1
		Sa34						9.8	12.6		35.6	24	
		Sa35						10.9	11.4	19	36.9		
		Sa36-36bis				22.8				18.6	35.1	25.5	20.3
		Sa37							9.3			21.4	
		Sa45									32		
		Unspecified									31.6		
		Sa47											19
		Sa41				25.5							
		Sa24				20.5							
Morlo et al. (2019a, b) (African data)	*A. giganteus*	CUMW 53					10.0	15.0	20.0	25.5	38.4	28.5	
		NHM M 82373b								15.7	28.2	19.3	
		PQAD 1520f								21.3	35.5	26.5	
Peigné et al. (2006a, b)	*A. giganteus*	LB-20											21.3

Measurements from the Afyon-1 specimen were compared with specimens from Ginsburg and Antunes (1968), Peigné (2012), Morlo et al. (2019a, b), Peigné et al. (2006a, b). The inventory number is given unless not stated; otherwise, the museum or locality where the specimen originates from is stated. All measurements in mm

3.
